# Allele, phenotype and disease data at Mouse Genome Informatics: improving access and analysis

**DOI:** 10.1007/s00335-015-9582-y

**Published:** 2015-07-11

**Authors:** Susan M. Bello, Cynthia L. Smith, Janan T. Eppig

**Affiliations:** Mouse Genome Informatics, The Jackson Laboratory, Bar Harbor, ME 04609 USA

## Abstract

A core part of the Mouse Genome Informatics (MGI) resource is the collection of mouse mutations and the annotation phenotypes and diseases displayed by mice carrying these mutations. These data are integrated with the rest of data in MGI and exported to numerous other resources. The use of mouse phenotype data to drive translational research into human disease has expanded rapidly with the improvements in sequencing technology. MGI has implemented many improvements in allele and phenotype data annotation, search, and display to facilitate access to these data through multiple avenues. For example, the description of alleles has been modified to include more detailed categories of allele attributes. This allows improved discrimination between mutation types. Further, connections have been created between mutations involving multiple genes and each of the genes overlapping the mutation. This allows users to readily find all mutations affecting a gene and see all genes affected by a mutation. In a similar manner, the genes expressed by transgenic or knock-in alleles are now connected to these alleles. The advanced search forms and public reports have been updated to take advantage of these improvements. These search forms and reports are used by an expanding number of researchers to identify novel human disease genes and mouse models of human disease.

## Introduction

Modern genome sequencing technology has transformed human molecular genetics. Exome and whole genome sequence data from patients with diseases of unknown etiology have provided several hundred new candidate genes for many diseases. Comparison of gene, phenotype, and genetic disease data from mouse models aids in refinement of candidate genes, and thus, in identifying causative mutations and potential therapeutic targets. To enable use of mouse model data in translational research, the Mouse Genome Informatics (MGI, www.informatics.jax.org) resource offers access to integrated mouse data, including experimentally and computationally generated data sets, to correlate mouse phenotype with human clinical signs and symptoms (Eppig et al. 2015a). MGI catalogs all mouse mutant alleles, annotates genotypes of mice carrying mutations to standardized phenotype terms and Online Mendelian Inheritance in Man (OMIM, www.omim.org) human disease descriptions, and includes links to other supporting gene information including sequence, polymorphism, spatiotemporal expression, genomic location, biochemical function and process, subcellular topology, and mammalian gene homology. Standardization of nomenclature and annotation of data with bio-ontologies and standardized vocabularies ensure that data are consistently annotated, making precise data mining possible.

Data provided by studies of the laboratory mouse have been shown to be valuable in driving discovery of human disease mutations. For example, a recent study describes the discovery of a variant of human *PNPLA8* that causes mitochondrial myopathy (OMIM 251950, Saunders et al. 2015). A mouse mutation in this gene with a similar phenotype had been previously described in Mancuso et al. 2009. Similarly, a mutation in the human *FAT1* gene was recently shown to be causative for a facioscapulohumeral dystrophy-like disease (Puppo et al. 2015). Mice carrying a muscle-specific conditional mutation in *Fat1* or a constitutive hypomorphic allele were shown to develop muscular and non-muscular defects mimicking human FSHD and these studies predicted the discovery of the human mutation in the same gene (Caruso et al. 2013). In addition, mouse models can provide valuable insights into molecular mechanisms and therapies. For example, studies of mutations in the mouse *Trp53* gene have been used to infer functions of the human *TRP53* gene, the most frequently mutated gene in human cancer, and have led to key understanding of functions of this gene as well as its role as a therapeutic target (Donehower 2014). MGI continues to provide integrated access to mutations in mouse to aid such discoveries related to human disease.

MGI collects, integrates, and presents data describing published and contributed spontaneous, induced, and genetically engineered mouse mutations. Downloaded data from large-scale mouse mutagenesis projects, including *N*-ethyl-*N*-nitrosourea (ENU), gene trap, and knockout mutagenesis projects (Smith and Eppig 2012) are also incorporated into the MGI dataset. The phenotypic consequences of all of these mutations in mice are described using the Mammalian Phenotype Ontology (Smith and Eppig 2012) and associated with human disease terms from OMIM (Eppig et al. 2015a) to enable consistent searching and data retrieval across all mutation types. MGI currently holds (Table 1) over 43,000 alleles present in mice which have been used to investigate phenotypes and diseases in over 55,600 and 4500 genotypes, respectively. These genotypes have contributed to the understanding of phenotype and disease consequences of mutations in over 15,300 and 2000 genetic markers, respectively. These disease, mutation, and phenotype data are fully integrated with all other MGI data including genomic, expression, tumor, and pathway knowledge to enable complex queries across multiple data types.

**Table 1 Tab1:** Selection of counts of disease, mutant allele, and phenotype data in MGI (data compiled on May 13, 2015)

Mutant alleles in mice	43,026
Genes with mutant alleles in mice^a^	13,316
Transgenes and other complex mutations	7456
Genotypes with phenotype annotations^b^	55,620
Genotypes with disease annotations	4500
Markers with phenotype annotations^c^	15,335
Markers with disease annotations^c^	2042

^a^Includes unmapped phenotypic markers

^b^Includes annotations to no abnormal phenotype detected

^c^Includes unmapped phenotypic markers, chromosomal aberrations, and transgenes

Mouse phenotype data generated by the International Mouse Phenotyping Consortium (IMPC) is the newest addition to MGI’s automated phenotype data integration pipeline, with new data added weekly. This worldwide consortium plans to generate knockout mice using the embryonic stem cell knockout resource developed by the International Mouse Knockout Consortium (IKMC, Bradley et al. 2012). Mice made with these mutant alleles are subject to high-throughput phenotyping screens. Statistically robust phenodeviants are automatically assigned MP terms and these phenotyping calls are downloaded to MGI (Koscielny et al. 2014).

**Fig. 1 Fig1:**
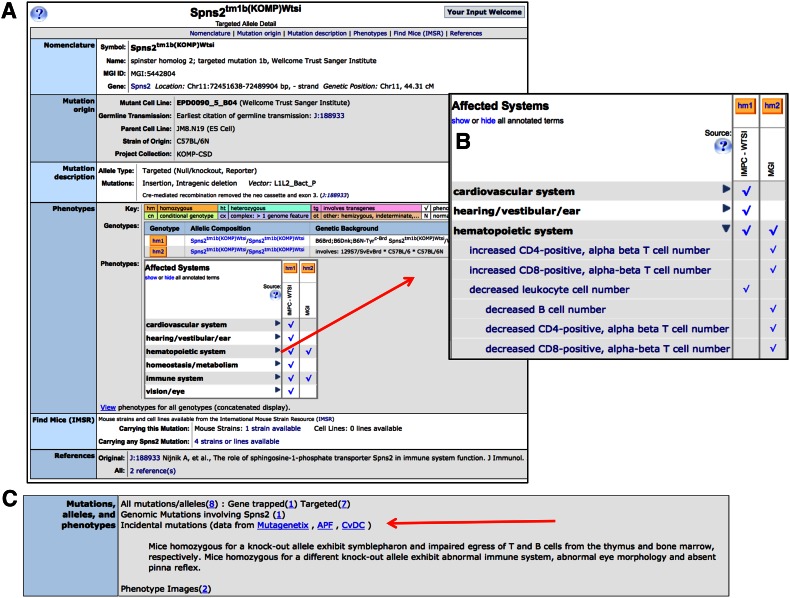
MGI allele detail page for *Spns2*
^*tm1b(KOMP)Wtsi*^ Information available includes **a** Nomenclature, mutation origin, project collection, and molecular details, when available, phenotype data, IMSR data for location of this mutation in a public repository, references. **b** Phenotype detail viewed by clicking on the toggle for “hematopoietic system.” **c** Section of the Marker detail page for Spns2 showing all known mutations for that gene, other genomic mutations including Spns2, incidental mutations found in genome wide sequencing, a short phenotype summary and links to image and disease data

Integration of these data with all other phenotypic data at MGI facilitates comparison of the effects of different mutation types to support comparative and correlative discovery and hypothesis building (Fig. 1a, b).

## Allele description features

### Nomenclature

Each allele is assigned a unique symbol according to nomenclature guidelines set by the International Committee on Standardized Genetic Nomenclature for Mice. Any synonyms, which may appear in the literature or other online resources, are added and are searchable in any nomenclature field. Users may submit new allele information to MGI (www.informatics.jax.org/mgihome/submissions/amsp_submission.cgi) to request or reserve nomenclature. Reserved alleles are kept private until publication unless the user requests that the allele(s) be made public.

### Mutation description

Alleles in MGI are described using a combination of free text and controlled vocabularies. We recently refined the description of alleles by introducing vocabularies for generation method and allele attributes. The generation method vocabulary includes the various techniques used to create new mutations (e.g., targeted, transgenic, endonuclease-mediated). Attributes include both functional changes to the endogenous gene and the function of inserted sequences. Endogenous gene mutation descriptors include knockdown, knockout, modified isoform(s), and no functional change. The terms constitutively active, dominant negative, hypomorph, humanized sequence, and conditional ready may be applied to either an endogenous gene mutation or an inserted sequence. The remaining terms (i.e., recombinase, reporter, transactivator) are all typically applied to inserted sequences. An allele may have multiple attributes attached but only a single generation method. While the term ‘inserted expressed sequence’ can overlap with multiple other attributes (i.e., recombinase, transposase, reporter, transposon concatemer), it is only used when the inserted sequence does not fit within another attribute. The Phenotypes, Alleles & Diseases query form enables you to search by generation method, attribute, or combinations of these (see Advanced Query Forms below).

### Project collections

Many alleles in MGI are contributed as part of a large dataset. These allele collections are assigned a project collection name and annotated to the alleles in the set. For example, the *Spns2*^*tm1b(KOMP)Wtsi*^ allele is part of the KOMP-CSD (Knockout Mouse Project, consortium of CHORI (Children’s Hospital Oakland Research Institute), the Wellcome Trust Sanger Institute, and the University of California at Davis) collection. Collections can be searched from the Phenotypes, Alleles and Diseases query form in conjunction with other allele data.

### Phenotype and molecular images

**Fig. 2 Fig2:**
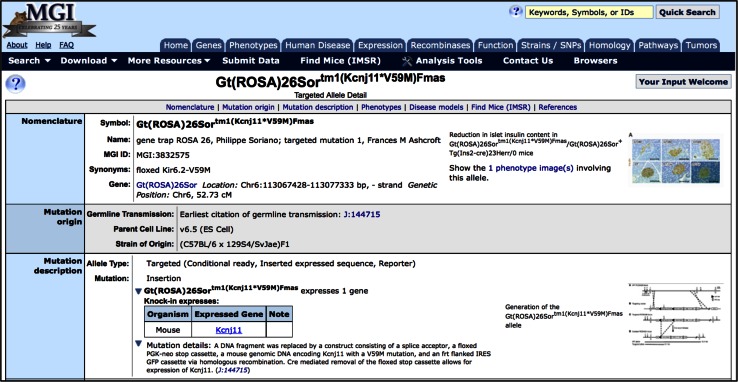
MGI allele detail page for *Gt(ROSA)26Sor*
^*tm1(Kcnj11*V59M)Fmas*^ shows annotation of the expressed component of this knock-in allele and a molecular image showing integration details. A mutated form of the mouse Kcnj11 gene is expressed from the endogenous Gt(ROSA)26Sor promoter after Cre-mediated recombination

Many alleles are made using complicated constructs. Displaying a diagram of the construct in many cases enhances the ability of users to understand the design of the allele compared to a description of the allele alone. Molecular images are now displayed for alleles when MGI has permission to display the image. Images and video can also enhance understanding of phenotypes. MGI displays phenotype images submitted by researchers and from publications where the journal has granted permission to display these images (Fig. 2).

## Incidental mutations

During large-scale mouse mutagenesis screens, especially using chemical mutagens, multiple mutations can occur in the genome for any given founder line. Modern sequencing methods have uncovered novel mutations in these mutant mouse lines that may not be of primary interest to the researcher conducting the screen. MGI has a listing of additional uncharacterized mutations discovered by sequencing in these mutant lines of mice. These data may be accessed from gene detail pages or from allele detail page molecular description. For example, the gene *Spns2* has incidental mutation data, accessible from the “Mutations, alleles, and phenotypes” ribbon of the marker detail page (Fig. 1c). These data may be useful to a researcher looking for novel mutations in a particular gene.

## Phenotype annotations

### Phenotype matrix

The phenotype matrix view presents a mutant allele in all of its studied contexts, whether it is a single gene mutation studied in one or more genetic strain backgrounds, or more complex genotypes including conditional genotypes, multigenic mutations, transgenic models, or combinations of these. The Genotypes table above the matrix provides the allele composition and strain genetic background for each mouse. Within the matrix rows the Genotype symbols (e.g., hm1, ht2, etc.) may be used to open a more detailed view that includes phenotype terms, notes, and links to references. This link opens in a separate page to facilitate comparison of multiple genotypes. Within the matrix, a high level summary term in the left column may be toggled to reveal more detailed phenotype terms (Fig. 1b) for comparisons among genotypes. Clicking on a checkmark within the matrix opens the same detailed view as the Genotype symbol and places the relevant term at the top of the window. There is a link under the matrix that opens a window showing the full text annotations for all genotypes in the matrix listed sequentially.

### Mammalian phenotype ontology

Phenotype descriptions at MGI are provided using the Mammalian Phenotype Ontology (MP, Smith and Eppig 2012). The MP contains over 10,000 phenotype terms, arranged in a complex hierarchy. The MP is used for phenotype annotation of all mutation types, enabling consistent descriptions and full search retrieval results for phenotype data. For example, the *Spns2*^*tm1b(KOMP)Wtsi*^ allele, homozygous 2 genotype is annotated with ‘decreased B cell number.’ Clicking on this term will open the MP browser, with links provided to retrieve all genotypes carrying any mutation annotated to this term or any subclass of this term. MP terms may also be used to search for mutations using the Phenotypes and Alleles Query Form, the Marker Query Form, for expression data and for disease model data and other portals (see below). Recent improvements to the MP include the addition of terms to support importation and integration of the high-throughput phenotyping data provided by Europhenome, Wellcome Trust Sanger Institute and the IMPC (Smith and Eppig 2015). This dataset is searchable along with all other MGI phenotype data.

## Disease annotations

The disease matrix includes published mouse models of a disease. If an attempt was made to create a mouse model of a disease but no phenotypic similarity was found this is also captured in the disease matrix. Clicking the Genotype symbols in this section opens the same full text annotation window as from the phenotype matrix. Clicking on a disease term name opens the MGI Disease Detail page. On the disease detail page the full set of mouse models in MGI for a disease may be found. The OMIM IDs link out to the OMIM website.

## Find mice (IMSR)

If an allele is available from a repository that participates in the International Mouse Strain Resource (IMSR), links to all strains carrying the allele are provided in the FindMice (IMSR) section of the Allele Detail page. In addition to links to strains carrying the exact allele, there is a link to all strains carrying any mutation in the same gene available from a participating repository (Eppig et al. 2015b).


## References

At the bottom of every Allele Detail page is a reference section. The Original reference, usually the first publication that reported the allele, is displayed here along with a link to a summary of all references annotated to this allele. From the reference summary you can access other alleles annotated from the same paper along with other types of data annotated in MGI from the reference.

Reference set links can be found at the bottom of most pages in MGI. We have recently added the ability to access the set of disease relevant references from the gene and disease detail pages. From the gene detail page these references are papers that include mouse models of a disease where the mutation in this gene is probably causative for the disease. This link includes all disease relevant papers for the gene and may include multiple diseases if mutations in the gene are causative for more than one disease. From the disease detail page the reference set includes all references annotated to this disease in MGI. In some cases, the mouse model used in the paper may not be fully annotated in MGI. All reference links go to the standard MGI reference summary where filters (author, journal, year, data) are available to further refine the list. The reference summary also allows users to export the reference list as a text file.

## Enhanced gene-to-allele connectivity

To increase access of alleles based on either the genes expressed by the allele or the genes overlapping the allele, we have added two new types of gene-to-allele relationships. For knock-in and transgene alleles we have created connections between the allele and the mouse gene or mouse homolog of the non-mouse gene expressed by the allele. With these connections, the transgene, Tg(APPswe,PSEN1dE9)85Dbo, which expresses human *PSEN1* and a humanized mouse *App*, can be accessed from the *App* and *Psen1* gene detail pages in MGI. The knock-in allele *Col1a1*^*tm1(hs1473*-*PITX1)Smun*^, which expresses human *PITX1*, can be accessed from the mouse *Pitx1* gene detail page.

**Fig. 3 Fig3:**
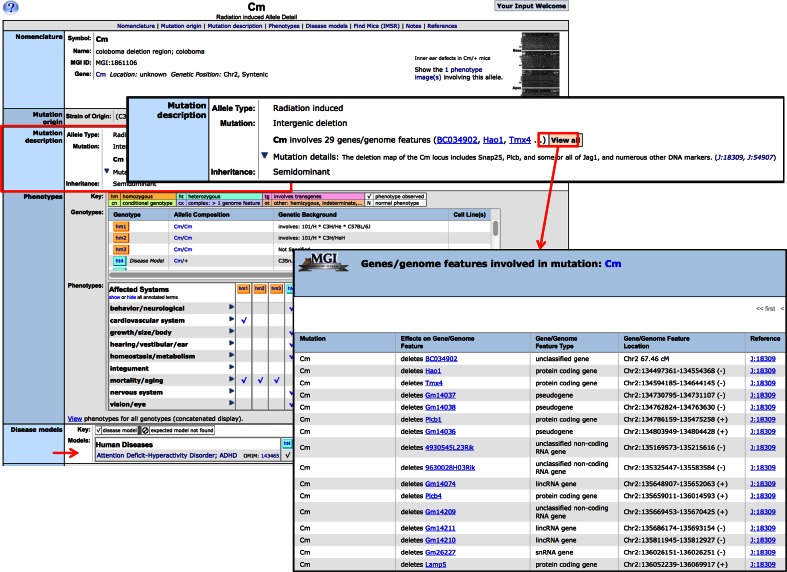
MGI allele detail page for *Cm* shows annotation of the endogenous genes mutated by this large deletion. In the mutation description section the first 3 genes are shown with a link that opens another window showing the full list of genes and additional information on how the gene is modified by the mutation. Note, at the *bottom* of the page is a section showing the human diseases modeled by mice carrying the *Cm* mutation

For alleles that overlap multiple genes, the new relationship type creates links between the allele and all genes that overlap the mutation region. The deletion region *Cm* overlaps 29 genes or genome features. The full list of features overlapping the region is accessible from the allele detail page and the allele can be found on each of the gene/genome feature detail pages that overlap the region (Fig. 3). This improved connectivity makes it easier to find all alleles related to a particular gene. These relationships are also used to improve the computation of gene-to-phenotype and gene-to-disease annotations from MGI’s corpus of genotype-to-phenotype and genotype-to-disease annotations (Bello et al. 2015).

## Finding data in MGI

### Advanced query forms in MGI

**Fig. 4 Fig4:**
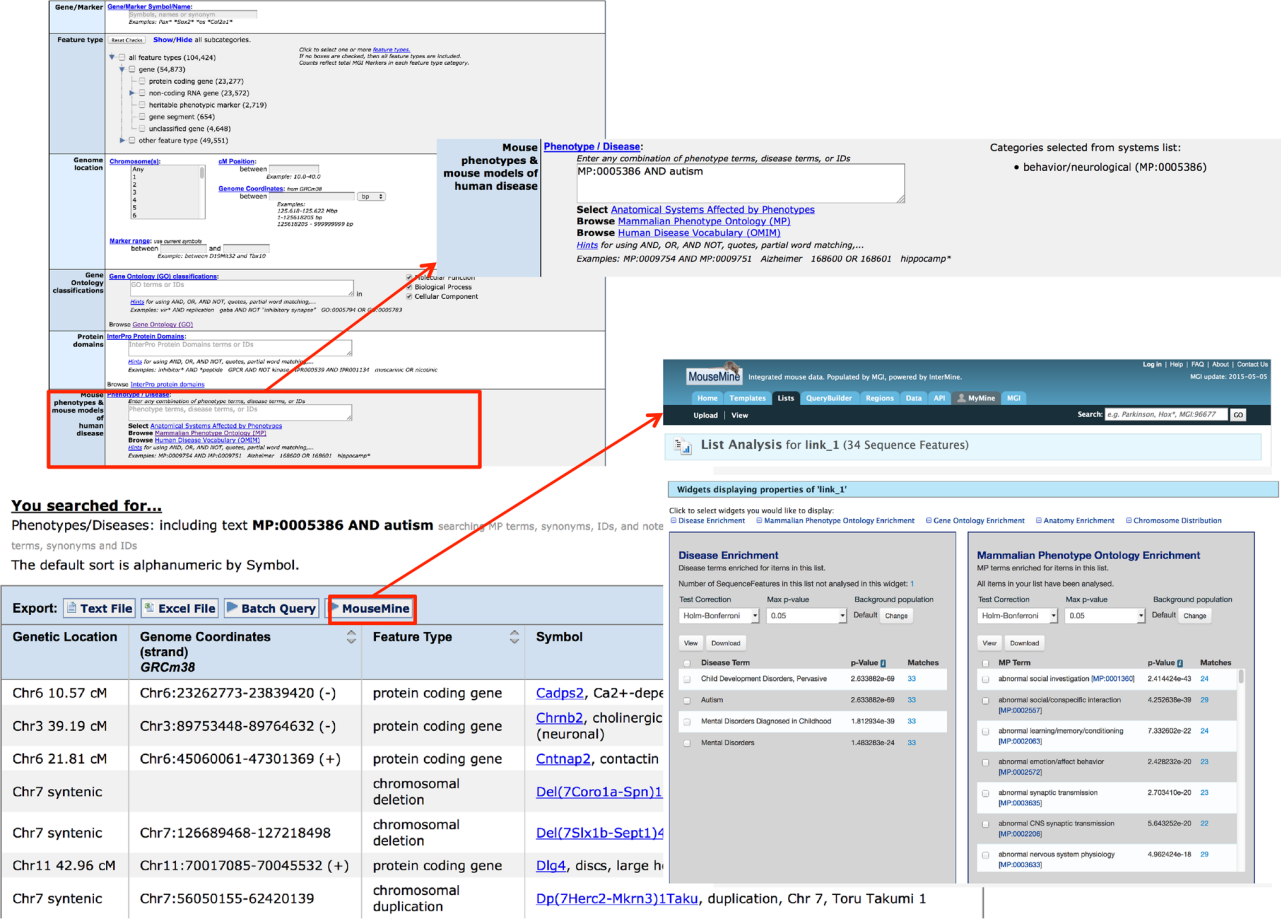
The MGI Genes & Markers Query now features an improved Phenotype/Disease search tool. When terms are selected from the “Select Anatomical Systems Affected by Phenotypes” tool the term name and ID are shown next to the search box. Searches may combine multiple MP or OMIM terms using Boolean operators. Results of a search may be downloaded, forwarded to the batch query, or exported to MouseMine

The phenotypes and diseases search box on the Genes and Markers and Phenotypes and the Alleles & Diseases query forms has recently been re-implemented to improve functionality. The search box now supports Boolean operators (AND, OR, AND NOT). The tool to select system-level MP terms has been converted to a pick list that adds the MP ID for the selected term to the query. The full name of the selected term is shown to the right of the search box (Fig. 4). Terms separated by spaces or commas are connected with a Boolean OR in the search. From both of these forms results can now be exported as a text file or excel file. From the Genes and Marker query form results may also be forwarded to the Batch Query form or to MouseMine (www.mousemine.org, Sullivan et al. 2013). For example, you can search for genes with mutations annotated to behavioral phenotypes that are also associated with Autism. Export the list of genes and sequence features returned by the query to MouseMine for further analysis (Fig. 4). MouseMine, built using the InterMine (Kalderimis et al. 2014) framework, allows users to conduct enrichment analysis on their gene set or build customized searches to gather more MGI data for the genes in their list.

**Fig. 5 Fig5:**
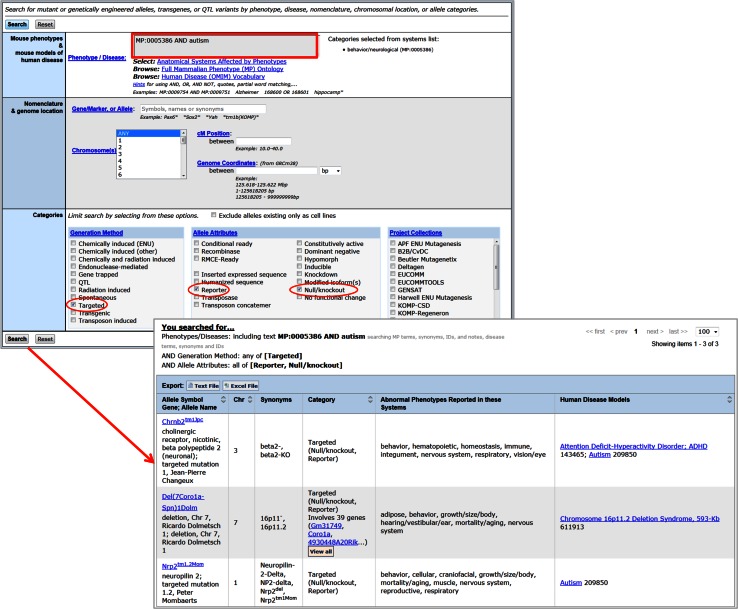
The MGI Phenotypes, Alleles & Disease Models Search now features an improved Phenotype/Disease search tool and the ability to search by allele Generation Method, Attributes, and/or Project Collections. In the Phenotype/Disease search box MP and OMIM disease terms may be connected with Boolean operators as on the Genes & Marker Query. Searches can combine terms in the Phenotype/Disease search box with selected Generation Method and/or Allele Attributes. Results of the search may be downloaded as a text or Excel file

In addition to the improved text search box, the Phenotypes, Alleles & Diseases query now supports searching for alleles based on generation method, attributes, and/or project collection. This is done by selecting one or more checkboxes found at the bottom of the query form. When multiple checkboxes are selected the choices are connected with Boolean ANDs. Thus, searching for alleles annotated to behavior and autism and selecting generation method “Targeted,” allele attributes “Reporter,” and “Null/knockout” will return all targeted alleles in MGI that are known to be knockouts, have a reporter gene inserted in the locus, and are annotated to the desired phenotype and disease terms (Fig. 5).

### Quick search

The ability to find alleles using the quick search tool has been improved. A search for using a synonym for a gene now returns the gene plus all alleles of the gene. This occurs even when the allele record is not directly annotated to the synonym. For example, a search for *SIN1* will now return the gene *Mapkap1* for which *SIN1* is a gene synonym and also will return all alleles of *Mapkap1*. Only one allele of *Mapkap1* (*Mapkap1*^*tm1Bisu*^) has an allele synonym that includes *SIN1* (*SIN1*^−^). Prior to this improvement, only the gene and this single allele would have been returned when a search for *SIN1* was executed from the quick search box.

**Fig. 6 Fig6:**
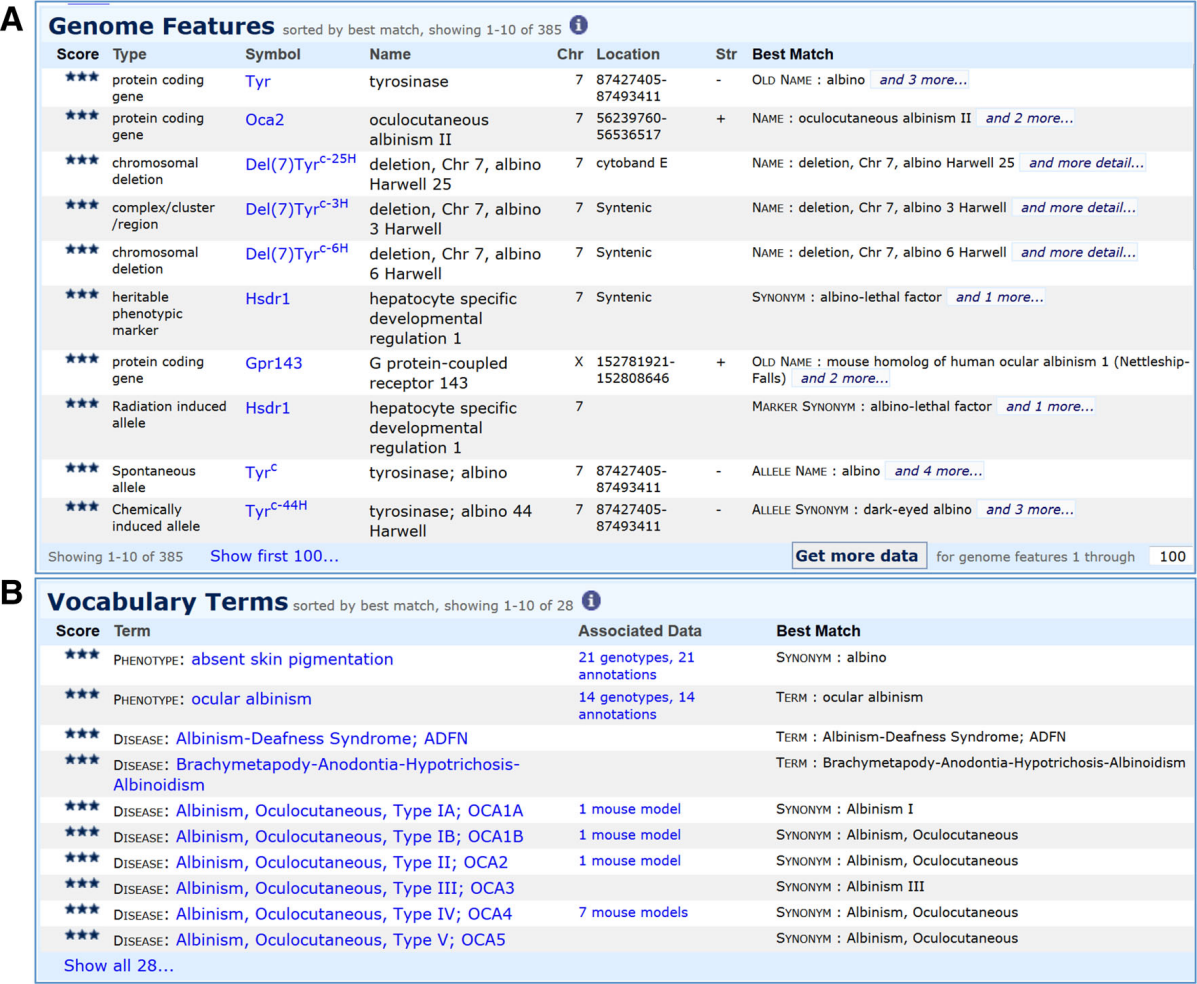
Results of an MGI quick search for “albin*”. **a** The Genome Features section includes both genes and alleles where the symbol or name matches the search and genes and alleles that are annotated to a matching vocabulary term. **b** The Vocabulary Terms section includes links to matching MP, OMIM, GO and other vocabulary terms along with links to annotations to each term in MGI

When words entered in the quick search box match an MP or OMIM disease term the alleles annotated to the term(s) are shown in the first section of the results. In the second results section, MP and OMIM disease terms matching the search are shown. These results link to an MP or Disease detail page where you may view more information about the term and access all annotations to the term. For example, a search for “albin*” will return in the first section genes with a matching synonym (i.e., albino is a former name for tyrosinase, *Tyr*) and alleles with a matching synonym or that are annotated to a matching vocabulary term (Fig. 6a). In the second section, the MP terms “absent skin pigmentation” and “ocular albinism” along with multiple OMIM disease terms are returned (Fig. 6b).

## Use of phenotype data outside of MGI

The Mammalian Phenotype Ontology and MGI’s phenotype annotation dataset have been incorporated into a myriad of tools used for discovery (Smith and Eppig 2012). More recently described tools and web services include MetaRanker, a web server for prioritization of common and rare frequency genetic variation data (Pers et al. 2013); Manteia, an online resource that compares embryological, expression, molecular, and etiological data from human, mouse, chicken, and zebrafish to predict the function of human genes (Tassy and Pourquié 2014); PhenomeNET 2, a system that enables similarity-based searches over a large repository of mouse, rat, and human phenotypes (Hoehndorf et al. 2015); PhenoDigm, a tool which integrates phenotype data from a variety of model organisms to identify gene candidates for human genetic diseases (Smedley et al. 2013); DisGeNET which prioritizes gene-disease associations through integrated data from expert-curated databases, text-mined data, and phenotype data from animal disease models (Pinero et al. 2015); and The Monarch Initiative which seeks to integrate biological data across multiple species for knowledge discovery (www.monarchinitiative.org).

Using these tools or other datasets derived from MGI, many research groups have discovered novel genes or pathways relevant to human disease studies. Using Manteia described above, Abath Neto et al. (2014) found multiple candidate genes for monogenic myopathies using datasets including mouse phenotypes. Honti et al. (2014) used semantic similarity of MP annotations and several other data types to develop a phenotypic network of genes. This network was then used to identify functional convergence among three sets of autism genes from independent exome studies.

In addition to gene discovery, gene function may also be inferred from phenotype data. Function may be inferred from semantic similarity of ontology terms and function may be deduced from relationships among phenotype terms when they arise as a result of one gene function. Ascensao et al. (2014) developed methods to infer 4818 unique GO functional predictions for 1796 genes.

## Summary/future plans

New sources of mouse phenotype data are anticipated in the coming year and MGI is planning to integrate and make available this new information. For example, CRISPR/Cas9 (Clustered Regularly Interspaced Short Palindromic Repeats/CRISPR associated system) (Sung et al. 2012; Singh et al. 2014; Aida et al. 2015) technology to generate germline mutations is now being used by researchers and MGI already has included a few examples from direct data contributions and the published literature (e.g., Concepcion et al. 2015, and unpublished data submissions.). The IMPC is planning to generate a significant number of lines via this technology (INTRAFRONTIER Consortium 2015) and we are preparing to incorporate these allele and phenotype datasets into the corpus of MGI data.

MGI also plans to incorporate new QTL and strain model data including data from the Collaborative Cross (Threadgill and Churchill 2012) and the Diversity Outcross (Churchill et al. 2012) projects. Analysis of mouse strains produced by these projects have already provided new genetic loci relevant to human conditions (Phillippi et al. 2014; Chesler 2014; Recla et al. 2014). While MGI already represents QTL data, improvements to the display in a genomic context, annotating data with the Vertebrate Trait Ontology (Park et al. 2013) and search improvements are planned or are underway.

MGI continues to work to improve access to mouse and human comparative data to support clinical and translational research. A mapping of terms between the Mammalian Phenotype Ontology and the Human Phenotype Ontology (Köhler et al. 2014) is ongoing, and will enable comparison between phenotype characteristics of mutant mice and signs and symptoms of humans with genetic-based disease. We will use these data to improve portals and searches to find appropriate mouse models, to narrow a set of potential candidate genes for a human condition or to find further genomic, biochemical process or function, pathway, expression, or location information about a specific gene product for greater understanding of a gene’s role in pathogenic processes in human disease.
